# Extra Supplement.—The Nursing Mirror

**Published:** 1888-11-10

**Authors:** 


### The Nursing Mirror.

All Contributions for this Supplement should be addressed "The Nursing Mirror," care of The Editor, 38, Old Jewry, E.C.

### En Passant.

THE MATRON OF LONGTON.—Many of our readers will be glad to learn that Miss Ashdown has recovered from her long illness, and is going to try the effect of three weeks' holiday. Miss Ashdown feels that she will not now be equal to moving to the new Cottage Hospital when it is built, and has therefore sent in her resignation, much to the regret of the committee.

CHURCH ARMY NURSES.—There is this to be said for the Church Army, that it trains its nurses. On October 24th Miss Constance Warner addressed the meeting at the Savings Bank, Wrexham, in aid of the Mission Nurses' Home. She explained to an attentive and interested audience how the nurses were trained and what work they did. The use of military terms was strictly adhered to throughout the lecture.

JUBILEE NURSES IN SCOTLAND.—There is a very sensible little note in *Truth* about the absurdity of starting a Scottish branch of the Queen Victoria Nurses' Institute on the £300 which is their share of the fund. A further appeal to the public to start the work makes the fact of this being supposed to be the Queen's gift to her people utterly ridiculous. It would be far better to give the money to the excellent training schools which already exist in Edinburgh and Glasgow.

A SUGGESTION.—We have received the following sensible letter to which we gladly give prominence, though the words "menial work" when used by a nurse always arouse a certain amount of wrath. We have never encountered work, which, if done by a noble woman, could be truthfully called menial. "A short time ago I was seeking for an engagement, and I saw the following advertisement: 'Wanted, a hospital-trained nurse, to take entire charge of an elderly lady, slightly paralysed.' I applied, and obtained it; but found, when I got there, that I was expected to do all kinds of menial work. I should like to make a suggestion to those who may want nurses to act in that capacity. When advertising, to advertise for one who will not object to make herself generally useful; and I feel sure it would be more satisfactory to employer and employed.—A TRAINED NURSE."

NURSES IN VIENNA.—If the account of the Vienna Hospitals and Nurses in this month's *Sunday at Home* were not from the pen of Mrs. Brewer, it would be difficult to believe it a faithful picture. Mrs. Brewer visited three hospitals—first, the Allemeines Krankenhaus, which holds 2,000 beds, and where there is a staff of 250 nurses taken from the streets and with no matron to superintend them. The hospital was dirty and disorderly, and offers a field for reformation, if only a Florence Nightingale could be found to undertake the work. The next hospital was the Rudolph Stiftung, which holds 850 beds, and is nursed by Catholic sisters and lay nurses. It seems a peculiar arrangement, which cannot give satisfaction or promote harmony. The third hospital described is the Rudolfinerhaus, nursed by the Red Cross Sisters: and all our correspondents, who so often make inquiries about this noble band of workers cannot do better than read Mrs. Brewer's article. Perfect order and discipline seem to reign under the Red Cross, and a probationer has to serve three years before the coveted badge is strapped round her left arm. How two such different hospitals as the last and first described by Mrs. Brewer can exist in the same town it seems impossible to imagine. It is time that nursing became the fashion in Vienna as it is in England and Germany, and then, perhaps, we might hope for a better state of things.

RELIGION AND NURSING.—Various letters have appeared in the *Tablet* in answer to Miss Maddon's sweeping assertion that Roman Catholics are not taken as nurses in the London hospitals. One writer says she has known a dozen Catholic nurses who have trained at St. George's without any difficulty being made about their religion, and some sisters of a Roman Catholic order have also been trained there. At the London and at Barts many Catholics have been trained, and at the first have been promoted to become sisters. Of course certain of the London hospitals are more sectarian than others, but considering the large proportion of Protestants in London, Catholics need not complain of this.

FRENCH SISTERS OF CHARITY.—The following particulars of nursing in Paris since the expulsion of the Sisters of Charity from the hospitals have been forwarded to us: "Yesterday the Court had before it a case of unparalleled debauchery, in which a hospital nurse of the new kind figured. The Judge said to her, 'You are a nurse in the Hospital of St. Louis, and you pass your nights in beer saloons.'"—*La Français.* In the month of April, 1884, two paid nurses were condemned to two months' imprisonment for having almost killed a patient who sought to prevent them from stealing wine. In the month of January, 1885, a paralytic, who occupied bed No. 19 in the ward of St. Francois, in the Hospital of Beauyon, was dragged from his bed by a drunken nurse and thrown into the cellar, where he expired a few minutes later. Dr. Chalvan, on December 22nd, 1884, wrote: "The evil that has been inflicted upon the hospitals is even greater than I care to say. Order and morality have been banished from our hospitals ever since the Sisters were expelled." It is only fair to our readers to remind them that these facts all belong to a few years ago, and that a state of transition is always one of more or less unavoidable disorder and difficulty. The latest reports from France bespeak a daily improvement in nursing.

THE MARCH OF PROGRESS.—In every quarter now the nurses seem to be receiving more consideration. At Grimsby the Hospital Committee have organised a system of "home nurses," who are sent to private houses in the district for moderate payment, and so far it has worked admirably, mainly owing to the zeal and efficiency of the young women who are trained to the duties. At Worcester it has been decided to make a change in the nursing staff, so that in future each ward would have one nurse and one probationer, with a night nurse and assistant nurse in addition. There is also a course of lectures to be delivered to the nurses, and a recreation-ground for all the hospital inmates is to be laid out. At Bridgewater Infirmary the whole of the nurses (both day and night) are now all trained and certificated. This is a great improvement upon the old *régime*, and has afforded much satisfaction to the medical men, and tended to the comfort and welfare of the patients. At Taunton a Nursing Institute has been opened, which we describe this week. The Chairman of the Enfield Cottage Hospital, on the occasion of the opening of the new Victoria Wing, spoke thus of the nursing: "The sisters, who had been so many years devoted to their charitable calling, were beloved by everybody, and more particularly by the patients; for they gave their lives and energies to the sick. They never hesitated to give up their own rooms when patients came in, and to submit to serious inconvenience if thereby they could lessen the sufferings of others. They attended to their duties in the most excellent manner, and he could not speak too highly of the way in which the two sisters had carried on the work of the hospital." All these are signs of progress.

### Original Articles.

### LOQUACITY.

THE absolute necessity for physicians to keep silent regarding the secrets which patients give to their care, is so fully acknowledged that communications made to them are regarded as almost as sacred as those made under the seal of the confessional. No doctor, though tried and tempted to the uttermost, ever gossips in public over his cases, or reveals the knowledge he has gained of family skeletons. But a few nurses have yet to learn that for the sake of their profession they also must acquire the habit of maintaining silence as profound as the grave about the private affairs of their patients. It is well known that amongst all classes of women the tendency to loquacity is a constant source of evil, and many a home is ruined through gossip exchanged over a cup of tea. A small book lately published called "The Autobiography of a Slander" had a design of a snake in a teacup on its cover, and the aim of the story was to show how a careless, exaggerated statement led to tragic results. To keep a watch over our mouths is a hard lesson to learn, but one which is absolutely imperative for a nurse. Most rules for those about to engage in private nursing conclude with this sentence: "Nurses are most earnestly charged to hold sacred the knowledge which, to a certain extent, they must obtain of the private affairs of such households or individuals as they may attend"—and that the warning is needed is proved by many a physician. An American doctor writing lately on this subject said: "Loquacity induces the bad habit of entertaining one patient with what has happened to another: not to speak of the undesirable one of worrying and irritating your charges by 'too much sound of the human voice.' Not long since an eminent physician, in recommending a nurse to me very highly, concluded by saying, 'And, doctor, she knows how to say *just enough!*' Of another I was told, 'Oh, yes, she understands how to nurse; but she is a perfect word-spout.'"

Most of us know a few word-spouts who, somehow or other, have managed to secure positions as nurses, and whose flow of language is a worry, not only to the poor patient, but to the whole establishment in which they are located. One such woman went by the nickname of "The Brook," because it was popularly reported that she could "go on for ever."

An Eastern tale is told of a hakim, who, coming forth from the Sultan's palace, was asked, "How does the Sultan?" "The Sultan sleeps," he replied. In one hour from that time he was exiled from Turkey, and told that he had just escaped the bowstring because he had dared to tell the secrets of the Sultan's palace to the world! Learn as little as you can of the domestic affairs of households into the bosoms of which you are admitted, and this little keep within your breasts, shut up as secrets entrusted to your honour!

It may seem a little thing to quarrel with nurses because they chatter while at tea, but the habit of continual talking cannot be indulged in without some resource to the imagination, or an enlarging on everyday facts. And a nurse must remember under what peculiar circumstances she comes into contact with human nature; when, stripped bare of its conventionalities, all that exalts or debases a soul in its hour of pain and trial is exposed to the gaze of a stranger. The mystery of birth, the solemnity of death, the dismay of the sufferer, the devotion of love, the depths of despair—all these are witnessed constantly by a nurse, but should never be made the subject of chatter or letters. It is better for a nurse's mind, it is better for her reputation, that these things should be considered sacred, and entombed in her heart. Throughout all countries the doctor is looked upon as the "flower of our civilisation, the possessor of generosity such as is possible to those who practise an art, never to those who drive a trade; discretion tested by a hundred secrets; tact tried in a thousand embarrassments, and what are more important, Heraclean cheerfulness and courage." These are the words of Mr. R. L. Stevenson, but are merely the expression of the common opinion. And when will nurses secure a reputation for tact and discretion as well as for kindness and skill? Perhaps you say that reputation is already won, and are indignant with the assumption that nurses are guilty of betraying professional secrets; but, alas! if many are careful and discreet, some are still talkative and imprudent. It is no sin to like to exchange comments about cases, but it is a dangerous amusement, like skating on the edge of a precipice; from the state of the patient's temperature one is apt to descend to the state of the patient's temper, and the discussion of his behaviour leads to an account of his relatives' behaviour and their reasons for doing as they do. Before we are aware of the danger the mischief is done, and we have shown to another the pitiful naked skeleton we have discovered in the private closet of the household where we have been nursing. And can anything be more contemptible, more treacherous, than to betray the secrets of a stricken family who have turned trustfully to us in their hour of sorrow? We who see men and women at their weakest, when they have no longer any strength to conceal their bitter scars, ought to cover up these marks of old wounds with as much care as though they were our own. How easily does conversation, which is the cream of companionship, if carried on too long become sour and turn to gossip. That nurse who knew how to say "just enough" was a remarkable specimen of womanhood. As a rule nurses are not nearly so much given to gossip as other women, for having much to do they have little time to talk; but their special temptation lies in this, that their work is often all-absorbing to them and their conversation includes little else, and then from talking of their work they imperceptibly drift into particulars about the patients on whom it is wrought. The nurse in a private family, in her desire to find cheeerful subjects for conversation, is sometimes led to make fun of former families, and indulge in highly-coloured descriptions of her various experiences. The world is very small though, and it has happened that a nurse has ridiculed the friends of the family she was with, and thus placed herself in a most humiliating and uncomfortable position quite inconsistent with the dignity of her profession. A hasty glance at the daily papers, an effort made to find time to read a new book or a magazine article, will be repaid by giving a nurse some thoughts outside the narrow groove in which she too often chooses to move.

It is to Carlyle we owe the great respect in which silence is frequently held; it was he who pointed out that one of the great dangers of the age lay in its loquacity, its tendency to reduce all matters either human or divine to a mere matter of words. We cannot do better than conclude this short paper by a quotation from his works: "To him that will well consider it, idle speaking is precisely the beginning of all hollowness, halfness, want of faithfulness, the genial atmosphere in which rank weeds of every kind attain the mastery over noble fruits in man's life, and utterly choke them out. Wise, of a wisdom far beyond our shallow depth, was that old precept 'Watch thy tongue;' out of it are the issues of life. Man is properly an incarnated word; the word that he speaks is the man himself. Was the tongue suspended there that it might truly tell what we had seen, and make man the soul's-brother of man; or only that it might utter vain sounds, jargon, soul-confusing, and so *divide* man, as by enchanted walls of Darkness, from union with man. Consider the significance of *silence*, it is boundless, never by meditating to be exhausted, unspeakably profitable to thee! Cease that chaotic hubbub, wherein thy own soul runs to waste, to confused suicidal dislocation and stupor: out of silence comes thy strength. No idlest word thou speakest but is a seed cast into Time, and grows through all Eternity."

### Everybody's Opinion.

*[Correspondence on all subjects is invited. No communications can be entertained if the name and address of the correspondent is not given, or unless one side of the paper only be written on.]*

NATIONAL PENSION FUND FOR NURSES.—In THE HOSPITAL of the 13th inst. I see that the Lincoln Hospital nurses were reported to have been present at the meeting at St. Thomas's to hear papers read on the subject of the Pension Fund. This is a slight error, as none of my nurses were present.—C. M. BEACHCROFT, Matron, County Hospital, Lincoln.

[Miss Beachcroft will see on reference to No. 107 Vol. V. of THE HOSPITAL for October 13th, that the statement is "Several delegates were present from provincial hospitals, Lincoln and Gloucester being especially well represented, but, through a misunderstanding, they did not speak." We have made careful inquiry and we find our statement is correct, the representative of the Lincoln nurses having wished to speak but being prevented because no card was sent up to the chairman. —ED. *T. H.*]

THE PRIVATE NURSE.—I think that in many cases a private nurse would not be expected to take her meals with the servants were it not for a clause in the rules of many nursing homes (it was of the home to which I belonged), which states that the nurse is to be treated as a trusty upper servant. That is all very well when you are in attendance in a nobleman's family, as surely no nurse could object to take her meals in the house-steward's or housekeeper's room; but as I used to find it the exception rather than the rule to be stationed in a first-class establishment, I often thought that such a rule was a mistake, inasmuch as it seemed likely to give rise to the unpleasantness of which "A Nurse who has found it a rule" complains. It was quite by accident I became aware of it, but on mentioning it to nurses who had lived in other homes, I found that mine was not an isolated case. On the other hand, when a private nurse is required, the household is generally upset by illness, perhaps a beloved child grappling with some dire disease, or a dear parent hovering between life and death, that it often happens we are neglected inadvertently. Should we not be willing to put self aside for a time, and be less particular as to what is due to our position and calling—those employing us will soon discover that for themselves. No doubt there are faults on both sides; how often one hears "Oh, I dreaded having a private nurse: a friend of mine had one and she wanted too much waiting on, she made the servant double work." I think if nurses would more readily adapt themselves to their oft-times difficult surroundings they would find their work easier, and their services more thoroughly appreciated.—T. A.

NURSE ANNA writes:—"In answer to the question in this week's HOSPITAL with reference to private nurses dining with the servants, I say it s a distinct insult to the profession at large that nurses should consent to take their meals in kitchen or servants' hall. My experience of private nursing has not been very large. In only one instance have I been asked to go to the servants' hall; on my refusing to do so my meals were served in a small sitting-room, the lady of the house observing, 'My sister-in-law has a nurse from —— Institution, London, who takes all her meals with the servants, which is very convenient.' Consequently I lost the case ——. It is to be feared that some nurses lower the standard which ought to be ours, by doing whatever the patient's friends may demand, just "to save a bother." If all institutions would make rules to protect the dignity of those employed by them, and those working on their own account would be equally firm—as the public must have good nurses—in time the educated, thoroughly competent nurse would compel respect, and meals in the kitchen be unheard of."

DORIS also writes:—"With reference to the paragraph in your last issue concerning a private nurse's position, I have never been asked to take my meals with the servants; nor in the kitchen. excepting in two small houses where the family did so themselves. Of course I need not say under these circumstances I made no objection, but under any others I should. I think a great deal depends on the nurse herself."

NURSES AND PRESENTS.—May I be allowed to make a few remarks to a letter in this week's HOSPITAL as regards the River Street Institute, Bath? I was a nurse there for a year, and I can only say, whoever the writer may be, he or she has not spoken correctly about the nurses' salary. We were paid from £25 to £30, and uniform, which is as much as a nurse gets in any institution. As to not being allowed to take presents, we certainly were not allowed to take money, but any other little present our Superintendent was only too pleased to see by it that her nurses were appreciated; and as to being sent away if they were ill after about a month or six weeks, I myself could mention the names of two nurses who were taken care of for more than three months, and were sent to the seaside at the expense of the Institution to regain their health. One of those nurses is onw working for the Bath Institution. The Bath Nurses' Home is a home for nurses in every sense of the word. I think there are few Homes where nurses are kinder treated, both by Superintendent and Committee. The late Superintendent (Miss Landale), although quite young, was in every sense of the word a mother to her nurses. A nurse was always sure of a kind welcome from her, and at any time a good word of advice, and her successor (Miss Taylor) is, if possible, equally as good.—A NURSE WHO WAS ONCE AT BATH. Nov. 2nd, 1888.

HOME FOR NURSES.—I am very pleased that "A Norwich Nurse" has spoken about a Home for Overworked Nurses in our large towns. There are Homes where governesses and servants can have complete rest after hard work; but if a nurse is ill or worked up, she must go to her friends, if she has any. I hope you will give us a suggestion. I shall anxiously watch your paper for one.—A MANCHESTER NURSE.

I THINK "A Norwich Nurse" cannot have read THE HOSPITAL with great care, or she would know that this question of a holiday Home for Nurses was discussed before and found faulty. Nurses want a thorough change when they are away from their work; they want the companionship of cheerful friends, who will cause them to forget their wards for a time. They don't want to be herded together in an institution to talk over each other's cases. Miss Phillippa Hicks' plan of sending her nurses to private houses, where a hostess makes friends with them, is simply perfect.—A LINCOLN NURSE.

### Appointments.

THROAT AND EAR HOSPITAL.—Miss Mackie, late night-sister at the London Hospital, has been appointed matron of the Throat and Ear Hospital, Golden Square. Miss Mackie was at one time matron of the Lock Hospital at Colchester, which is now closed, so that she has had experience in the duties she is about to undertake.

SHERBORNE INFIRMARY.—Miss Clarke has been appointed matron of this Infirmary. Miss Clarke trained at St. Bartholomew's, and has lately acted as sister at the Royal Naval Hospital, Haslar.

CHILDREN'S HOSPITAL, EDINBURGH.—The matronship of the Royal Hospital for Sick Children, Edinburgh, has been filled by the appointment of Miss E. J. R. Landale. Miss Landale was trained at Westminster Hospital, and was for more than three years lady superintendent of the Bath Trained Nurses' Home, which she successfully organised, and where she started district nursing before she left.

### Vacancies.

**The following vacancies are announced:—**

**Matrons.**

Aldershot Lock Hospital; £50.
Sherburn Hospital, Durham; £40.

**Nurses.**

Southport Infirmary; £20.

Eastern Fever Hospital, Homerton; £30.

Aldershot Lock Hospital; £30.

Kidderminster Infirmary; £20.

Chester Diocesan Deaconess' Institution; two.

South Devon Hospital.

Cardiff Infirmary; £24.

Home Hospital, Eastbourne.

Swansea Nursing Institute.

Bolton Infirmary; £30.

Bridgwater Infirmary; £22.

Croydon Nurses' Institute: several.

Newport and County Infirmary, Mon.; £25.

Royal Hospital for Children and Women, Waterloo Bridge Road, S.E.

Newcastle-on-Tyne Nurses' Home; several.

Harley Street Establishment; £25.

Barrow-in-Furness Hospital; £26.

Mold Cottage Hospital.

Cray Valley Cottage Hospital.

Hampshire Nurses' Institute.

West Bromwich District Hospital £25.

Nursing Institution, 96, 97, 98*a*, Wimpole Street, London, W., Mr. Wilson has several vacancies for thoroughly-trained Nurses.

Derby Children's Hospital.

Kingston Nurses' Home; several.

Samaritan Free Hospital for Women and Children, Lower Seymour Street, Portman Square.

Cheltenham Nursing Institution; several.

Bristol Hospital for Sick Children and Women; £20.

**District Nurses.**

Newcastle, Staffordshire.
Newlands, Ryde.

**Probationers.**

Stamford Infirmary.
Macclesfield Infirmary.
Royal Hospital for Children and Women, Waterloo Bridge Road, S.E.
Boston Hospital, Lincolnshire.
Bristol Royal Infirmary.
Brighton Borough Fever Hospital; £16.

### Notes and Queries.

**Queries.**

(78) *Caps.*—Where can materials for caps like those worn by Bartholomew's nurses be bought?—*Nurse E.*

**Answers.**

(76) *The Gate Porter and Female Visitors.*—"Reader" appears to be under an entire misapprehension. We have made inquiries of the authorities of the institution in question, and no complaint has been made to the Board as to the porter having been rough to a female visitor for a slight, or indeed for any, offence of the kind.

(77) *Medical Dictionary.*—K. G. A. is informed that Hoblyn's Dictionary is published by Whitaker and Co., at 12*s.* 6*d.*

### Inquests on Babies.

The deaths among babies are enormous, and especially among those which are artificially fed. A contemporary says: "At an inquest recently, in St. Pancras, on a body that had been improperly fed, Dr. Danford Thomas warned mothers and nurses against giving infants not a-year old the patent foods so extensively advertised. Most of these foods, he said, contained a good deal of starch; and as young babies cannot digest starch, they would waste away. Milk and water was the proper substitute for Nature's food for babies." These are timely words from Dr. Danford Thomas, for the people cannot be too well informed in matters of this kind. There is a great deal of nonsense in the old saw about "a little knowledge." Every fact is a golden apple of truth, which as many as possible should possess. That only priests should read theology and the Bible, and only doctors read medicine, is the relique of a flagrant error that in former times held sway.

Dr. Thomas deals a blow, and very properly, at the habit of feeding babies on artificial foods. Most of these are claimed to be equal to, or better than, mother's milk. It cannot be fully understood that they are nothing of the kind. As Dr. Jacobi says that children sometimes survive on them is no reason why they should be universally recommended, for infants thrive on many things. One feels that it is impossible to touch upon this vital theme too often, for it is one of ever-present importance. If starchy foods are bad, how comes it that they have such a sale? Such is the question of the self-styled practical man or woman. The reason is not difficult to guess. Poor milk, watered milk, cheesy milk, when mixed with starchy food, curdles less densely; for the presence of the latter throughout the fluid renders the casein, when clotted by the rennet-ferment of the stomach, more crumbly; so that the delicate organ is better apt to deal with it. By this step, then, some are nourished that without it must needs have succumbed from wasting and diarrhœa. The sugar present also enriches the poor milk. Accordingly, the inference has been drawn that starchy food was good for infants; and foods containing some 80 per cent. of starch are consequently advertised and largely sold. Another contributive cause, perhaps, is the fact that in diarrhœa occasionally, the administration of powdered biscuits will stop the ailment.

Bread-jelly, barley-water, gelatine, crushed and ground cereals, have now so established themselves as agents in infant-feeding, that they could account for a large share of deaths. It is by no means alleged that as much does not depend upon proper nursing and care as upon proper food. It *is* maintained that many artificial foods are accountable for an unnecessarily large infant mortality.

As to the proposition that "milk and water is the proper substitute for Nature's food for babes," it may be admitted that some infants appear to thrive on these, though physiological differences between milks and the practice of evil customs, both among milk dealers and consumers, point out the unmistakable need of something more than the addition of water to milk. The addition of water reduces the proportion of casein, but does not affect its digestibility much; nor does it overcome the acidity resulting from the hawking about the streets; neither does it take into account the brilliant results obtained by sterilization. The proposition may, therefore, with propriety be modified to read somewhat as follows:—

Cow's milk partially pre-digested with a Zymine (Fairchild) Peptonising Powder, boiled, and supplemented with a little sugar and cream (or Kepler Solution of Oil in Malt), is the proper substitute for Nature's "food for babes."

